# Inhibitory Activity of Shrimp Waste Extracts on Fungal and Oomycete Plant Pathogens

**DOI:** 10.3390/plants10112452

**Published:** 2021-11-13

**Authors:** Soumia El boumlasy, Federico La Spada, Nunzio Tuccitto, Giovanni Marletta, Carlos Luz Mínguez, Giuseppe Meca, Ermes Ivan Rovetto, Antonella Pane, Abderrahmane Debdoubi, Santa Olga Cacciola

**Affiliations:** 1 Laboratory of Materials-Catalysis, Chemistry Department, Faculty of Science, University Abdelmalek Essaadi, Tetouan B.P. 2117, Morocco; soumiaelboumlasy@gmail.com (S.E.b.); debdoubi@hotmail.com (A.D.); 2 Department of Agriculture, Food and Environment, University of Catania, 95123 Catania, Italy; federico.laspada@unict.it (F.L.S.); ermes.rovetto@hotmail.com (E.I.R.); apane@unict.it (A.P.); 3 Consorzio per lo Sviluppo dei Sistemi a Grande Interfase, CSGI, Viale A. Doria 6, 95125 Catania, Italy; n.tuccitto@unict.it (N.T.); gmarletta@unict.it (G.M.); 4 Department of Chemical Sciences, Università degli Studi di Catania, Viale A. Doria 6, 95125 Catania, Italy; 5 Laboratory of Food Chemistry and Toxicology, Faculty of Pharmacy, University of Valencia, Av. Vicent Andrés Estellés s/n, 46100 Burjassot, Spain; carlos.luz@uv.es (C.L.M.); giuseppe.meca@uv.es (G.M.)

**Keywords:** metabolites, phenolic compounds, inhibitory effect, citrus, apple, HPLC-ESI-MS-TOF, post-harvest diseases, mal secco disease, MIC, MFC

## Abstract

(1) Background: This study was aimed at determining the *in vitro* inhibitory effect of new natural substances obtained by minimal processing from shrimp wastes on fungi and oomycetes in the genera *Alternaria*, *Colletotrichum*, *Fusarium*, *Penicillium*, *Plenodomus* and *Phytophthora*; the effectiveness of the substance with the highest *in vitro* activity in preventing citrus and apple fruit rot incited by *P. digitatum* and *P. expansum*, respectively, was also evaluated. (2) Methods: The four tested substances, water-extract, EtOAc-extract, MetOH-extract and nitric-extract, were analyzed by HPLC-ESI-MS-TOF; *in vitro* preliminary tests were carried out to determine the minimal inhibitory/fungicidal concentrations (MIC and MFC, respectively) of the raw dry powder, EtOAc-extract, MetOH-extract and nitric-extract for each pathogen. (3) Results: in the agar-diffusion-assay, nitric-extract showed an inhibitory effect on all pathogens, at all concentrations tested (100, 75, 50 and 25%); the maximum activity was on *Plenodomus tracheiphilus*, *C. gloeosporioides* and *Ph. nicotianae*; the diameters of inhibition halos were directly proportional to the extract concentration; values of MIC and MFC of this extract for all pathogens ranged from 2 to 3.5%; the highest concentrations (50 to 100%) tested *in vivo* were effective in preventing citrus and apple fruit molds. (4) Conclusions: This study contributes to the search for natural and ecofriendly substances for the control of pre- and post-harvest plant pathogens.

## 1. Introduction

Plant pathogenic fungi are responsible for many serious diseases that affect agricultural productions both pre- and post-harvest. In this respect, the losses of products along the post-harvest chains (i.e., warehousing, transport and final distribution) determine strong impactful consequences, especially in agriculture-based-economy countries [1,2,3]. To minimize production losses and maintain crop sustainability, several strategies based on the application of different means, such as physical, chemical and biological, have been adopted over time [4,5]. Currently, one of the most consolidated and effective means for controlling fungal diseases is represented by chemical synthetic fungicides [4,6]. However, their use negatively affects both human health and the preservation of the environment. Moreover, the restricted number of active ingredients which are allowed for post-harvest treatments increases the risk of selection of fungicide resistant plant pathogens, with the consequent dramatic reduction of the efficacy of synthetic fungicides [7]. For these reasons, during past years, their application has been strictly limited by several governmental institutions worldwide [8,9]. 

In order to satisfy the growing request for high-quality and, at the same time, safe and eco-friendly products, throughout the past two decades, the research field strongly focused on the investigation of the potentialities of alternative means to synthetic fungicides to control plant diseases; these include antagonistic microorganisms or derivatives thereof, natural biostimulants [7,9,10], as well as natural antimicrobial compounds [11,12].

With the perspective of reducing environmental pollution and related consequences for human health, nowadays, the scientific research is also strongly focused on valorizing wastes, especially those largely generated by processing industries [13]. Within this framework, the shrimp market has stood out for considerable development, especially during the past few years. In this respect, it has been estimated that in 2020, the production of shrimp reached a total of 5.03 million tons around the globe, with an amount of waste ranging between 40–50% per ton of fresh product [14,15,16]. Therefore, the wastes generated by shrimp processing industries in food production are clearly undergoing a dramatic increase [17]. Shrimp wastes generated for production of human food are represented by heads, intestines, tails and shells [17], which are usually disposed by throwing into garbage heaps [18], ocean dumping, incineration and land filling [19]. Therefore, an inevitable increase in generated wastes could be determined by their non-use [20].

Shrimp are, overall, considered a high-value aquaculture product [17], not only because of the nutritional properties of the meat used for human consumption, but also for the composition of their wastes; in fact, their major constituents are proteins (35–50%), chitin (15–25%), calcium and phosphorus (10–15%), and other substances (such as amino acids, vitamins, carotenoids, astaxanthin, polyunsaturated fatty acids and other enzymes) [15,21,22,23]. For this reason, nowadays, the valorization of shrimp wastes is a consolidated practice.

Shrimp wastes as such have been used for feeding in veterinary practice and aquaculture [17] as well as in compost fertilizer [24,25]. Dried shrimp wastes are also used in animal feeding in mixtures with other agricultural raw materials; however, since drying processes are usually carried out directly along the beaches, these practices of the use of shrimp wastes favor additional pollution, especially in coastal areas [17]. A further strategy for the use of shrimp wastes includes both the extraction of bioactive molecules or the secondary chemically-mediated transformation of some parts of these into other bioactive compounds; one of these is the chitosan, the large-scale production of which is commonly carried out by alkaline deacetylation of the chitin extracted from shrimp shells [26]. Chitosan has several useful applications in various fields, including medicine, cosmetics, agriculture, paper and textile industries, biotechnologies and bioremediation of the environment (water treatment) [15,27]; however, the acid/alkaline-mediated industrial processes for its production from shrimp wastes have serious environmental consequences [17,18,26].

The aforementioned products arising from shrimp wastes represent, therefore, a precious asset in several fields of application; however, it is an accepted fact that their processing generates highly impactful new wastes, which in turn contribute to environmental pollution and, consequently, negatively affect human health.

The investigation of the potentialities of new products arising from a minimal and sustainable processing of shrimp wastes stands, therefore, as an essential challenge for scientific research. Considering that plant pathology is strongly focused on finding eco-friendly strategies for controlling plant pathogens and related diseases, the present study evaluated the effectiveness of new substances obtained by the minimal processing of shrimp wastes in the *in vitro* and *in vivo* control of major fungal and oomycete pathogens of the genera *Alternaria, Colletotrichum, Fusarium, Penicillium, Plenodomus* and *Phytophthora.*

## 2. Results

**Figure 1:**
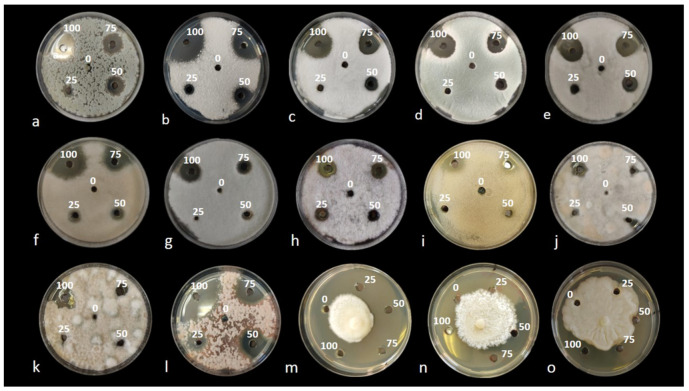
Agar diffusion test. Inhibition halos determined by the Nitric-extract at different concentrations (0, 25, 50, 75, 100%), after 3 days of incubation at 25 °C on PDA: (**a**) *Penicillium digitatum* P1PP0; (**b**) *P. commune* CECT 20767; (**c**) *P. expansum* CECT 2278; (**d**) *P. italicum* CECT 20909; (**e**) *Colletotrichum acutatum* UW14; (**f**) *C. karsti* CAM; (**g**) *C. gloeosporioides* C2; (**h**) *Fusarium proliferatum* CBS 145950; (**i**) *F. sacchari* CBS 145949; (**j**) *Alternaria arborescens* 803; (**k**) *A. alternata* 646; (**l**) *Plenodomus tracheiphilus* Pt2. Inhibition halos at different concentrations after 15 days of incubation at 25 °C on PDA: (**m**) *Phytophthora nicotianae* T2.C-M1A; (**n**) *Ph. nicotianae* T3-B-K1A; (**o**) *Ph. citrophthora* Ax1Ar.

In this study, wastes from the shrimp species Parapenaeus longirostris were processed to obtain four substances: (i) “Water-extract”, (ii) “EtOAc-extract”, (iii) “MetOH-extract” and (iv) “Nitric-extract”. All these extracts were analyzed, to determine their composition in metabolites and phenolic compounds, by HPLC-ESI-MS/TOF. Then, the antifungal activity of the “dry-powder”, “EtOAc-extract”, “MetOH-extract” and “Nitric-extract” was preliminarily tested *in vitro* by an agar diffusion test toward several fungal and oomycete pathogens. “Dry-powder”, “EtOAc-extract” and “MetOH-extract” did not demonstrate any inhibitory effect in the mycelial growth of all pathogens under study (data not shown); therefore, they were not further tested. “Nitric-extract” was the only extract that negatively affected the mycelial growth of all pathogens; the diameter of the inhibition halos consequently observed at each concentration was, therefore, recorded at the end of the incubation period (see Figure 1a–o). The most effective substance resulting from the *in vitro* test was further investigated to determine its efficiency in terms of minimal inhibitory concentration (MIC) and minimal fungicidal concentration (MFC). The *in vivo* effectiveness of the selected substance in the control of post-harvest infections of fruits by *Penicillium* spp. was finally tested.

### 2.1. Metabolites and Phenolic Compounds Detected in Test Substances by HPLC-ESI-MS/TOF

**Figure 2:**
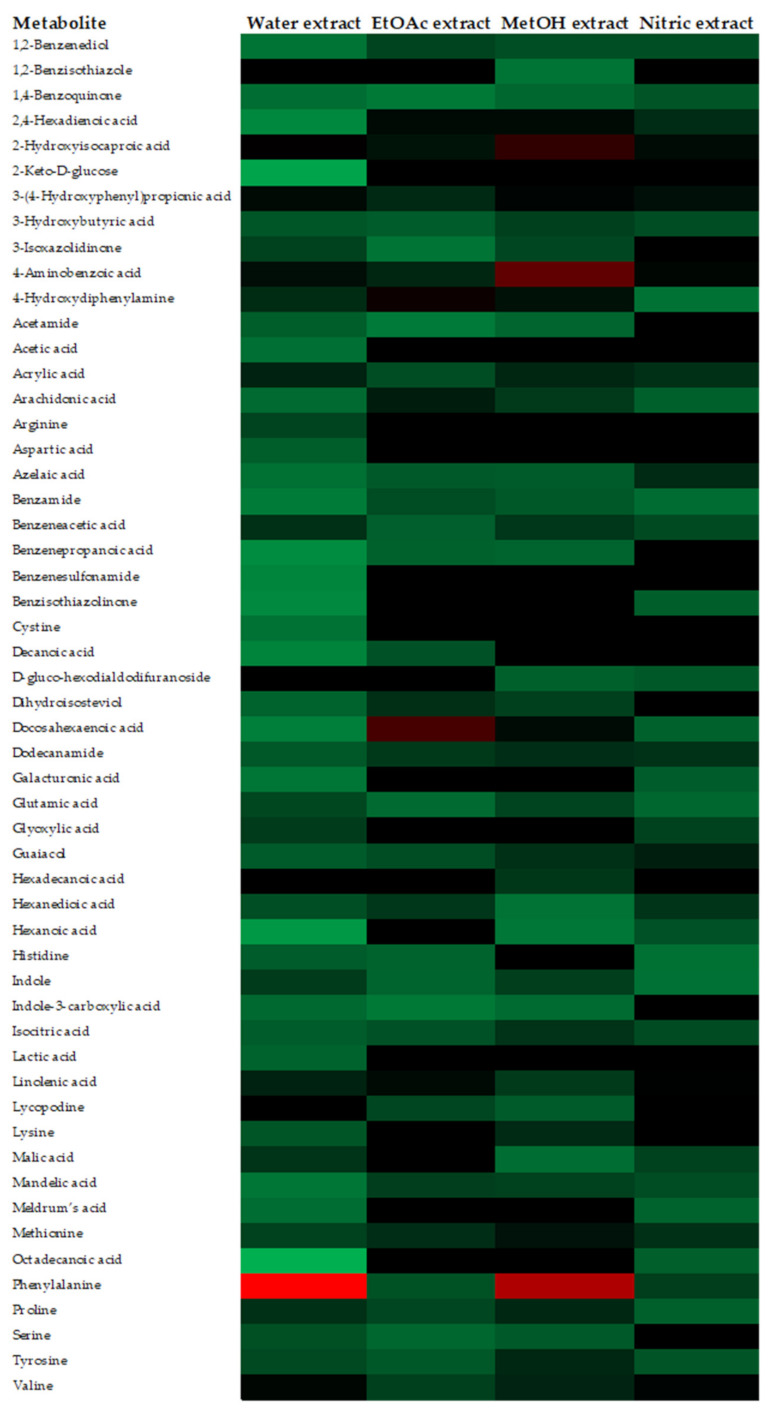
Heat map representing the relative abundances of metabolites detected in different shrimp extracts.

The metabolites detected by HPLC-ESI-MS/TOF in the analyzed substances are presented, as a heat map, in Figure 2. Colors are based on the relative abundance (logarithmic scale) of the metabolites detected, where red represents high abundance and green represents low abundance. Overall, among all substances examined, the analysis evidenced the presence of a total of 54 metabolites already known in the literature. In particular, the “Water-extract” showed 50 metabolites, which is the highest number recovered; “EtOAc-extract” and “Nitric-extract” contained 36 and 35 metabolites, respectively; finally, only 25 metabolites were detected in the “MetOH-extract”. Some marked differences were observed among the substances; in particular, a higher abundance of free amino acids, such as phenyalanine, proline, serine, tyrosine and valine, was evidenced in the “Water-extract” and “MetOH-extract” over the “EtOAc-extract” and “Nitric-extract”. Really high relative abundances in some metabolites were also observed; in particular, 2-Hydroxyisocaproic acid, 3-(4-Hydroxyphenyl) propionic acid and 4-aminobenzoic acid in “MetOH-extract”, docosahexaenoic acid in “EtOAc-extract” and the phenylalanine in the “Water-extract” and “MetOH-extract”.

The most important phenolic acids detected by HPLC-ESI-MS/TOF in the substances analyzed are presented in Table 1. Their abundance is expressed in mg/kg of each substance. The most abundant phenolic compound detected in all the analyzed substances was benzoic acid, whose amount ranged from a minimum of 0.87 mg/kg in “Nitric-extract” to a maximum of 3.57 mg/kg in “EtOAc-extract”. In order of abundance, vanillin (0.21–2.04 mg/kg) and syringic acid (0.16–1.21 mg/kg), which had the highest concentrations of “MetOH-extract”, were detected. The p-coumaric (4-hydroxycinnamic acid) acid was another phenolic compound recovered in all the substances; its abundance ranged from a minimum of 0.27 mg/kg in the “Water-extract” to a maximum of 0.88 mg/kg in the sample “Nitric-extract”. The “Nitric-extract” also reported the highest concentration of 1-2-Dihydroxybenzene (0.86 mg/kg). Few phenolic compounds were detected just in one substance; among these, the 3-(4-hydroxy-3-methoxyphenyl) propionic acid and ellagic acid were detected only in “Nitric-extract”, while sinapic acid only in the “Water-extract”.

### 2.2. In Vitro Preliminary Tests

Results from the *in vitro* preliminary tests evidenced an inhibitory effect on the growth of the pathogens examined only for the waste shrimp extracted with nitric acid, named “Nitric-extract”. Additionally, none of the control solutions (each solvent used for the preparation of the respective extract) inhibited mycelial growth. In the agar diffusion test, “Nitric-extract” at concentrations of 100, 75 and 50% showed an inhibitory effect on all strains of fungal and oomycete pathogens, while at concentration of 25%, an inhibitory effect was still observed only on *Ph. nicotianae* T2.C-M1A, *F. sacchari* CBS 145949, *A. alternata* 646, *P. digitatum* P1PP0, *P. commune* CECT 20767, *C. gloeosporioides* C2, *F. proliferatum* CBS 145950, *Pl. tracheiphilus* Pt2 and *Ph. nicotianae* T3-B-K1A, in order of significance (Table 2 and Figure 2). The diameter of inhibition halos was directly proportional to the concentration of the extract (Table 2). Significant differences in the inhibitory effects of the extracts were noticed among fungal and oomycete species as well as between species of the same genus and even between strains of the same species (Table 2). At the maximum dose, which is 100% of the extract concentration, the highest inhibitory effect was on *Pl. tracheiphilus* Pt2; at 75% concentration, the highest inhibitory activity was on *Pl. tracheiphilus* Pt2 and *Ph. nicotianae* T2.C-M1A; at 50%, on *Ph. nicotianae* T2.C-M1A; and at the lowest dose (25% extract concentration), on *Ph. nicotianae* T2.C-M1A as well as on three typically post-harvest pathogens, i.e., *F. sacchari* CBS 145949, *A. alternata* 646 and *P. digitatum* P1PP0.

### 2.3. Determination of MIC and MFC

To further test the inhibitory activity of “Nitric-extract” on the growth of pathogens, the minimum inhibitory concentration (MIC) and the minimum fungicidal concentration (MFC) were determined, and results are summarized in Table 3. The values of both MIC and MFC for all pathogens were in the range 2–3.5%. In more detail, the highest values of MIC (3.5%) were recorded for *P. expansum* CECT 2278 and *F. saccari* CBS 145949, while the lowest (2%) were recorded for *C. gloeosporioides* C2, *Ph. nicotianae* T3-B-K1A and *Pl. tracheiphilus* Pt 2. Values of MFC were the same as MIC for the majority of the strains. Only for strains *P. commune* CECT 20767, *A. alternata* 646, *Ph. nicotianae* T3-B-K1A and *Ph. citrophthora* Ax1Ar, MFC was higher than MIC, indicating that for these four strains, MIC exerted only a fungistatic effect.

### 2.4. In Vivo Antifungal Activity

The antifungal activity of “Nitric-extract” was finally tested *in vivo* on citrus (oranges and lemons) and apple fruits artificially infected by *P. digitatum* and *P. expansum*, respectively. Results are summarized below.

#### 2.4.1. Antifungal Activity on Oranges

**Figure 3:**
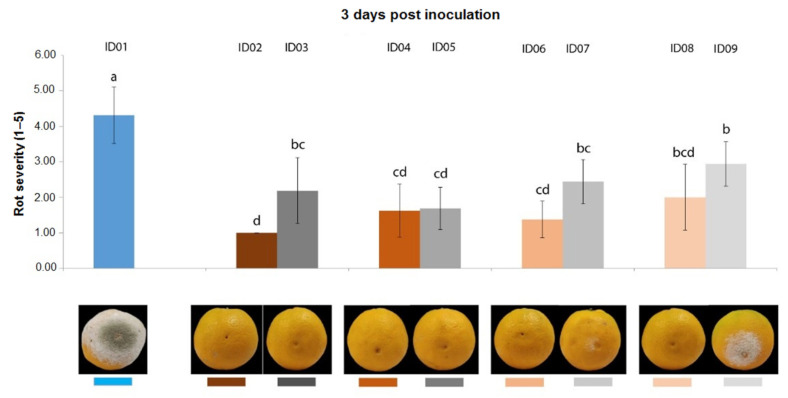
Rot severity caused by *Penicillium digitatum* strain P1PP0 in orange (*Citrus* × *sinensis*) fruits cv. Valencia treated with water (ID01) or nitric-extract as such (ID02), 75% Nitric-extract (ID04), 50% Nitric-extract (ID06), 25% Nitric-extract (ID08) and respective controls (NaNO_3_ 0.17 g/mL—ID03; NaNO_3_ 0.17 g/mL diluted in sterile distilled water (sdw) at 75%—ID05; NaNO_3_ 0.17 g/mL diluted in sdw at 50%—ID07; NaNO_3_ 0.17 g/mL diluted in sdw at 25%—ID09) 3 days after inoculation. Values sharing the same letters are not statistically different according to Tukey’s HSD (honestly significant difference) test (*p* ≤ 0.05). Bars represent SD.

Three days post inoculation with *P. digitatum* P1PP0 of oranges, all concentrations of “Nitric-extract” significantly reduced rot severity compared to the water control (treatment ID01) (Figure 3). However, except for “Nitric-extract” applied as such (ID02), each of the other concentrations was not statistically different from the respective control.

**Figure 4:**
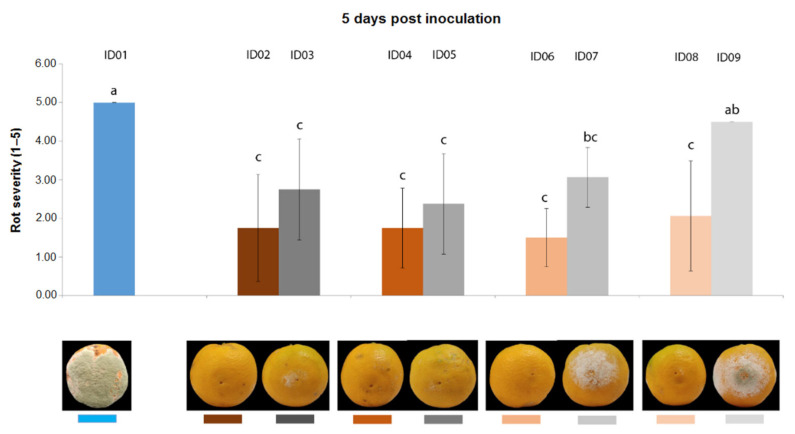
Rot severity caused by *Penicillium digitatum* strain P1PP0 in orange (*Citrus* × *sinensis*) fruits cv. Valencia treated with water (ID01) or nitric-extract as such (ID02), 75% Nitric-extract (ID04), 50% Nitric-extract (ID06), 25% Nitric-extract (ID08) and respective controls (NaNO_3_ 0.17 g/mL—ID03; NaNO_3_ 0.17 g/mL diluted in sterile distilled water (sdw) at 75%—ID05; NaNO_3_ 0.17 g/mL diluted in sdw at 50%—ID07; NaNO_3_ 0.17 g/mL diluted in sdw at 25%—ID09) 5 days after inoculation. Values sharing the same letters are not statistically different according to Tukey’s HSD test (*p* ≤ 0.05). Bars represent SD.

Five days after inoculation (Figure 4), all concentrations of “Nitric-extract” still demonstrated values of rot severity significantly lower than the water control (treatment ID01); however, in this case, only treatment with “Nitric-extract” at 25% (ID08) significantly differed from the respective control (ID09), although this difference was not statistically significant in comparison to the other control treatments (ID03, ID05, ID07).

#### 2.4.2. Antifungal Activity on Lemons

**Figure 5:**
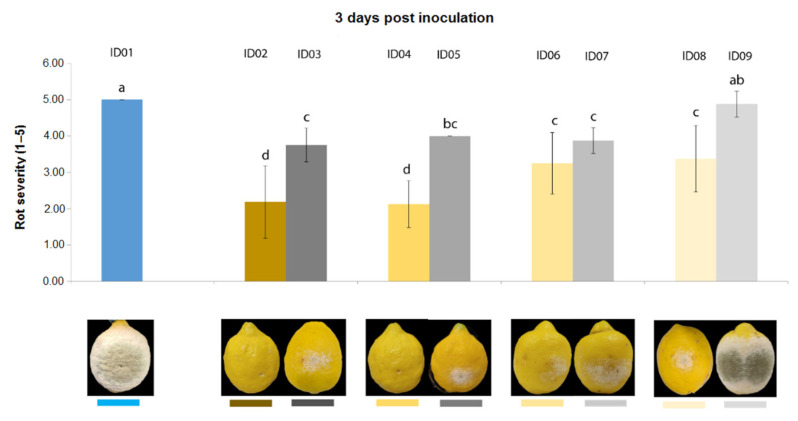
Rot severity caused by *Penicillium digitatum* strain P1PP0 in lemon (*Citrus* × *limon*) fruits cv. Femminello Siracusano treated with water (ID01) or Nitric-extract as such (ID02), 75% Nitric-extract (ID04), 50% Nitric-extract (ID06), 25% Nitric-extract (ID08) and respective controls (NaNO_3_ 0.17 g/mL—ID03; NaNO_3_ 0.17 g/mL diluted in sterile distilled water (sdw) at 75%—ID05; NaNO_3_ 0.17 g/mL diluted in sdw at 50%—ID07; NaNO_3_ 0.17 g/mL diluted in sdw at 25%—ID09) 3 days after inoculation. Values sharing the same letters are not statistically different according to Tukey’s HSD test (*p* ≤ 0.05). Bars represent SD.

**Figure 6:**
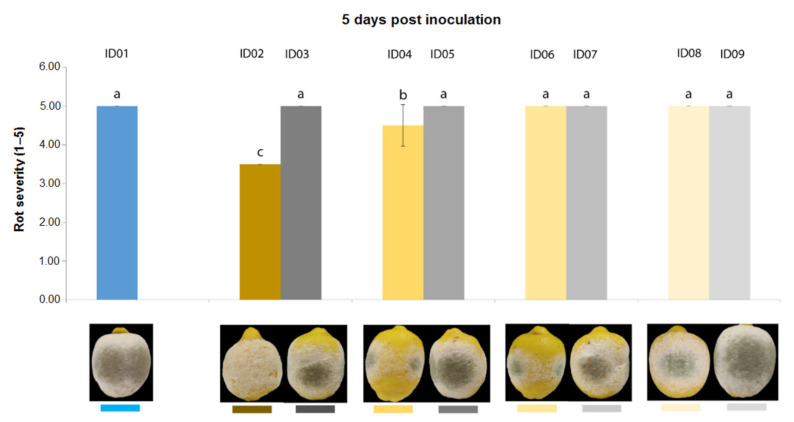
Rot severity caused by *Penicillium digitatum* strain P1PP0 in lemon (*Citrus* × *limon*) fruits cv. Femminello Siracusano fruits treated with water (ID01) or Nitric-extract as such (ID02), 75% Nitric-extract (ID04), 50% Nitric-extract (ID06), 25% Nitric-extract (ID08) and respective controls (NaNO_3_ 0.17 g/mL—ID03; NaNO_3_ 0.17 g/mL diluted in sterile distilled water (sdw) at 75%—ID05; NaNO_3_ 0.17 g/mL diluted in sdw at 50%—ID07; NaNO_3_ 0.17 g/mL diluted in sdw at 25%—ID09) 5 days after inoculation. Values sharing the same letters are not statistically different according to Tukey’s HSD test (*p* ≤ 0.05). Bars represent SD.

Three days after inoculation of *P. digitatum* P1PP0 in lemons (Figure 5), all tested concentrations of “Nitric-extract” significantly reduced rot severity compared to the control. Additionally, among these, “Nitric-extract” as such (ID02) and “Nitric-extract” at 75% (ID02) significantly reduced rot severity compared to all other control solution (treatments ID03, ID05, ID07 and ID09). “Nitric-extract” as such (ID02) and “Nitric-extract” 75% (ID04) were also the only treatments that, five days after inoculation, still maintained significant effectiveness in the reduction of rot severity in lemons (Figure 6).

#### 2.4.3. Antifungal Activity on Apples

**Figure 7:**
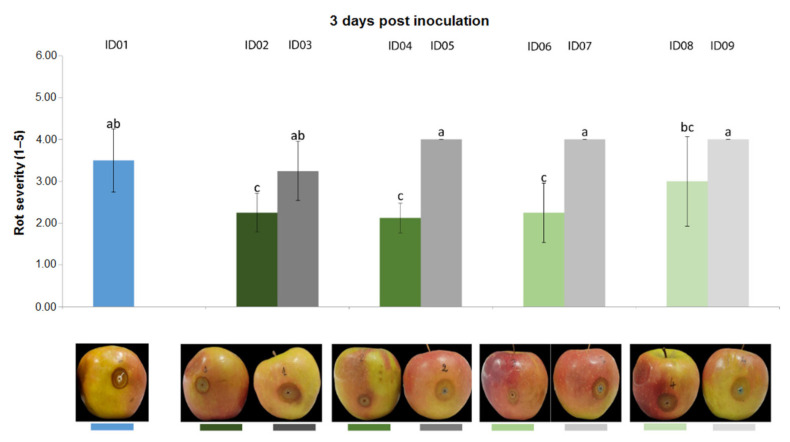
Rot severity caused by *Penicillium expansum* strain CECT 2278 in apple (*Malus domestica*) fruits cv. Braeburn treated with water (ID01) or Nitric-extract as such (ID02), 75% Nitric-extract (ID04), 50% Nitric-extract (ID06), 25% Nitric-extract (ID08) and respective controls (NaNO_3_ 0.17 g/mL—ID03; NaNO_3_ 0.17 g/mL diluted in sterile distilled water (sdw) at 75%—ID05; NaNO_3_ 0.17 g/mL diluted in sdw at 50%—ID07; NaNO_3_ 0.17 g/mL diluted in sdw at 25%—ID09) 3 days after inoculation. Values sharing the same letters are not statistically different according to Tukey’s HSD test (*p* ≤ 0.05). Bars represent SD.

**Figure 8:**
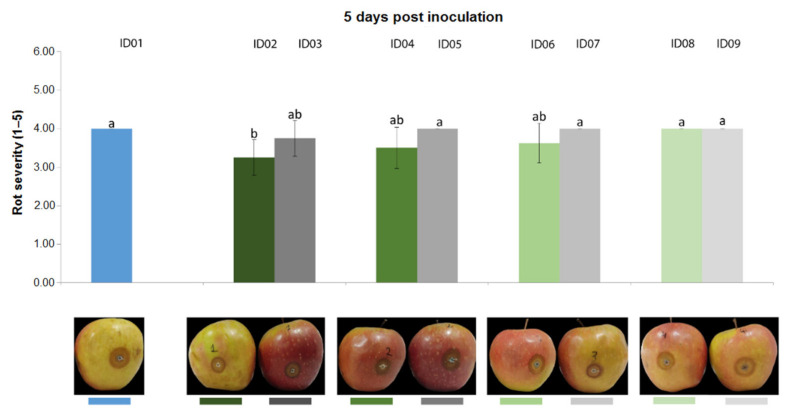
Rot severity caused by *Penicillium expansum* strain CECT 2278 in apple (*Malus domestica*) fruits cv. Braeburn treated with water (ID01) or Nitric-extract as such (ID02), 75% Nitric-extract (ID04), 50% Nitric-extract (ID06), 25% Nitric-extract (ID08) and respective controls (NaNO_3_ 0.17 g/mL—ID03; NaNO_3_ 0.17 g/mL diluted in sterile distilled water (sdw) at 75%—ID05; NaNO_3_ 0.17 g/mL diluted in sdw at 50%—ID07; NaNO_3_ 0.17 g/mL diluted in sdw at 25%—ID09) 5 days after inoculation. Values sharing the same letters are not statistically different according to Tukey’s HSD test (*p* ≤ 0.05). Bars represent SD.

Results from the trial carried out on apple fruits inoculated with *P. expansum* CECT 2278 evidenced that, three days post inoculation (Figure 7), “Nitric-extract” as such (ID02), at 75% (ID04) and at 50% significantly reduced rot severity in comparison with any other treatment and controls. Five days post inoculation, only “Nitric-extract” as such (ID02) still significantly reduced rot severity (Figure 8).

## 3. Discussion

This study evaluated, for the first time, the potentialities of minimally processed shrimp wastes in the *in vitro* inhibitory activity on fungal and oomycete plant pathogens, and their effectiveness in controlling post-harvest rots caused by *Penicillium* spp. in citrus and apple fruits. To this aim, wastes from the shrimp species *Parapenaeus longirostris* were dried and grounded to result in a “dry-powder”, which was further processed leading to four different extracts “Water-extract”, “EtOAc-extract”, “MetOH-extract” and “Nitric-extract”. Acid hydrolysis is mandatory for the mineralization of calcium-containing shrimp waste, and hydrolysis is commonly performed by hydrochloric, acetic, phosphoric, sulfuric, nitric and lactic acids. Nitric acid was selected as, among the above-mentioned acids, it has the slowest reaction kinetics [28], which allows for better digestion control. All these substances, “Water extract”, “EtOAc-extract”, “MetOH-extract” and “Nitric-extract, were analyzed to determine their composition in metabolites and phenolic compounds. Then, the “dry-powder”, “EtOAc-extract”, “MetOH-extract” and “Nitric-extract” were also preliminarily tested *in vitro*, in order to select the substance with the highest mycelial growth inhibitory activity. “Nitric-extract” was the most effective substance and was further investigated to determine its antifungal properties (in terms of MIC and MFC) and *in vivo* antifungal activity.

Results from the chemical analysis showed that all substances extracted from the shrimp waste were miscellaneous mixtures of a conspicuous number of metabolites and phenolic compounds. Interestingly, a high relative abundance of the 2-Hydroxyisocaproic, 3-(4-Hydroxyphenyl) propionic and 4-Aminobenzoic acids in “MetOH-extract”, and of docosahexaenoic acid in “EtOAc-extract” were reported. Various studies reported fungicidal activity for these molecules when tested as pure substances; 2-hydroxyisocaproic acid was effective against *Candida* and *Aspergillus* species [29]; 3-(4-Hydroxyphenyl) propionic acid contains the hydroxyl group, which has been reported as one of the substance responsible for the antifungal activity of *Lactobacillus paracasei* [30]. Moreover, the para-aminobenzoic acid showed antibiotic activity toward *Staphylococcus aureus* [31]; a *Pseudomonas aeruginosa-*bioconverted oil extract of docosahexaenoic acid was effective against the mycelial growth of several plant pathogens, including *Botrytis cinerea*, *Colletotrichum capsici*, *Fusarium oxysporum*, *F. solani*, *Phytophthora capsici*, *Rhizoctonia solani* and *Sclerotinia sclerotiorum* [32]. However, in the present study, two extracts, “MetOH-extract” and “EtOAc-extract”, containing a higher amount of the above-mentioned acids, showed no inhibitory activity on mycelial growth.

An additional interesting metabolite present in all substances was phenylalanine, which was also detected in high amount in “Water-extract” and “MetOH-extract”. A recent study [33] reported that post-harvest treatments of mango, avocado and citrus fruits with phenylalanine induced resistance against infections caused by *Colletotrichum gloeosporioides*, *Lasiodiplodia theobromae* and *P. digitatum*, respectively, although *in vitro* tests carried out in the same study evidenced no inhibitory effects toward the same pathogens. Therefore, although lacking of fungicidal action, the “Water-extract” and “MetOH-extract”, which showed a high amount of phenylalanine, could provide strong resistance induction properties to control post-harvest disease. It goes without saying that, since phenylalanine was also detected in “EtOAc-extract” and “Nitric-extract”, these samples could also have resistance induction properties, as demonstrated for other extracts of natural origin [34]. This possibility assumes a particular significance of the extract “Nitric-extract”, which was the only substance tested that demonstrated clear and strong *in vitro* antifungal activity as well as significant *in vivo* control of infective processes. Additional studies are, therefore, ongoing, to verify possible resistance induction properties of all the minimally processed shrimp wastes produced in this study. Quite interestingly, although the exoskeleton of shellfish is the main raw material for the extraction of chitosan, whose inhibitory activity on post-harvest fruit rots is well documented [35], this biopolymer was not present in the extracts examined in this study. As a consequence, it can be inferred that other substances are responsible for the antimycotic activity showed by the “Nitric-extract”.

With reference to composition in phenolic compounds, analyses evidenced the presence, in all tested substances, of molecules whose antimicrobial activity is supported by a wide range of literature [2,36,37,38,39,40,41,42,43,44]. Some of these compounds have been also applied as eco-friendly alternatives to synthetic fungicides [1,45]. Among the phenolic compounds, the molecules that recurred in all analyzed substances were the benzoic, caffeic and p-coumaric acids and the vanillin. Benzoic and caffeic acids have important preservative properties that determine the inhibition of fungal growth [43,46]. Vanillin (4-hydroxy-3-methoxybenzaldehyde) is considered one of the most important additives used in the food industry; it is characterized by effective inhibitory activity toward a wide range of microorganisms, thus causing a delay in the growth of yeasts and fungi [36,40]. The p-coumaric acid (4-hydroxycinnamic acid), which, in “Nitric-extract”, had the highest concentration, is the main phenolic acid contained in the peel of sweet oranges [44], and is well known for its efficacy in negatively affecting the growth of post-harvest pathogens, such as *Monilinia fructicola, Botrytis cinerea* and *Alternaria alternata* [2]. Interestingly, “Nitric-extract” also reported the highest concentration of catechol (1-2-dihydroxybenzene) and the exclusive presence of dihydroferulic (3-(4-hydroxy-3-methoxyphenyl) propionic acid) and ellagic acids. Catechol shows significant activity in the control of *Fusarium oxysporum* and *Penicillium italicum* [38]. Dihydroferulic acid significantly inhibits the *in vitro* growth of *Saccharomyces cerevisiae, Aspergillus fumigatus* and *A. flavus* [39]. Moreover, ellagic acid, which possesses well-documented antibacterial activity [37], shows extraordinary antifungal effects toward *Botrytis cinerea* [41], as well as a significant growth inhibition of several fungal species belonging to the genera *Trichophyton* and *Candida* [42]. Finally, phenolic compounds are hypothesized to be, at least in part, responsible for the strong broad-spectrum antifungal activity shown by a pomegranate peel extract [34].

Overall, unlike the “Water-extract”, “EtOAc-extract” and “MetOH-extract”, “Nitric-extract” results were characterized by p-coumaric acid and catechol, both present at high concentrations, and by the exclusive presence of the acids dihydroferulic and ellagic; these molecules could be, therefore, responsible for the antifungal activity of this extract. Synergetic action of some of the molecules detected in “Nitric-extract” also cannot be excluded. This effect has already been observed for the active components of extracts from different natural matrices. This is the case, for example, of pomegranate, whose high biological value is recognized as being the result of the synergistic chemical action of the total phytoconstituents of the fruit rather than of single extracted components [47,48,49].

The quantity and quality of the molecules that were active (individually or in synergy) in determining the *in vitro* antifungal activity of the tested substances could also be related to the extraction process. By comparing the compositions of the three extracts, namely, “EtOAc-extract”, “MetOH-extract” and “Nitric-extract”, the three applied extraction processes had different efficiencies. The choice of the best solvent for the extraction of precise bioactive components from a specific matrix is a crucial aspect for reaching the expected qualitative and quantitative yield of the desired molecules in the final extract [34]. Examples of this aspect are provided by studies carried out on pomegranate extracts; Al-Zoreky [50] observed that the 80% methanolic extract was richer in polyphenols compared to hot water and diethyl ether extracts and, therefore, led to higher antimicrobial activity against pathogenetic bacteria. Tayel et al. [51] found that, regardless the concentration of specific bioactive components, a methanolic pomegranate peel extract was more effective than ethanol and water extracts in controlling *Penicillium digitatum*. In view of these aspects, it is quite surprising that, among the extracts, only “Nitric-extract” provided *in vitro* antifungal efficacy and, at the same time, neither “EtOAc-extract” nor “MetOH-extract” resulted in an inhibitory effect on mycelial growth.

Results from the *in vitro* preliminary test together with those from MIC and MFC tests overall demonstrated that the pathogens mostly affected by “Nitric-extract” were *Pl. tracheiphilus* Pt 2, *C. gloeosporioides* C2 and *Ph. Nicotianae*—both tested isolates. *Plenodomus tracheiphilus* is the causal agent of ‘mal secco, one of the most destructive diseases affecting lemon trees [52]. Because of the vascular propagation of the pathogen in all aerial parts of the infected plant, the management of the disease is complicated [53]. It is commonly carried out by the pruning of diseased twigs, withered shoots and suckers, followed by the spraying of the canopy with copper-based fungicides, which can reduce the occurrence of new *Pl. tracheiphilus*-infections. However, many copper-based treatments are not cost effective in commercial lemon groves, and also represent a significant source of environmental pollution [53]. Another copper-susceptible pathogen is *C. gloeosporioides*, the causative agent of anthracnoses in several fruits and vegetables [54] as well as of twig and shoot dieback in citruses [55]. *Phytophthora nicotianae* is very likely the most widespread and destructive *Phytophthora* species worldwide, affecting a very wide host range of more than 255 plant species [8,56,57]. Control strategies may be different depending on the specific situation, although the pathogen is markedly sensitive to Metalaxyl and Fosetyl Al, fungicides which are commonly used for controlling plant diseases affecting roots, collars and stems [56]. Results from this study pose “Nitric-extract” as a promising alternative to the use of conventional fungicides in controlling not only *Pl. tracheiphilus*, *C. gloeosporioides* and *Ph. nicotianae*, but all pathogens tested in the present study. To this aim, further investigations are needed to evaluate the phytotoxicity, if any, of the extract, its attitude to systemic translocation, which is of particular relevance in the case of tracheomycoses, such as ‘mal secco’ caused by *Pl. tracheiphilus,* as well as the most effective method of application, e.g., by drenching, spraying or incorporation into fruit coatings, which also depends on the type of disease.

As a preliminary step towards the application of “Nitric-extract” to control plant diseases, its effectiveness was tested *in vivo* against molds caused by *Penicillium* species in orange, lemon and apple fruits, which are the most economically important post-harvest diseases affecting these fruits [58,59]. Post-harvest molds of citrus and apple fruits are traditionally controlled by the application of highly effective chemicals, such as imidazole and bendimidazole (thiabendazole) fungicides [60,61]. More recently, as a consequence of the selection of imidazole- and bendimidazole-resistant strains of *Penicillium*, several other synthetic fungicides, including azoxystrobin, fludioxonil, cyprodinil and pyrimethanil, have been proposed as alternatives for the chemical control of these post-harvest fruit diseases [7,60,61,62,63]. Like imidazoles and benzimidazoles, all these fungicides are effective at relatively low doses but are characterized by a high acute toxicity [64,65,66,67,68,69].

There is boundless literature evaluating the efficacy of alternative strategies to the use of conventional synthetic fungicides for the control of postharvest molds of *Penicillium* species [70,71,72,73,74,75,76,77,78]. A novelty in the present study is the *in vivo* control of *Penicillium* spp. using a natural substance that is derived from minimum waste treatment.

Overall, treatments with “Nitric-extract” at the highest concentrations were the most effective in positively affecting the reduction of rot severity in all tested fruits. Additionally, an interesting weak positive effect was also observed in all control treatments, including NaNO_3_ in water solution (ID03, ID05, ID07 and ID09), although, *in vitro,* they were not effective in inhibiting the mycelial growth of all pathogens included in this study. As already observed for other inorganic salts [74], it cannot be excluded that the *in vivo* effectiveness of NaNO_3_ was not the consequence of direct antifungal activity, but the possible result of the triggering of defense mechanisms in fruits. Further tests are ongoing to verify this hypothesis.

The results from the treatments with “Nitric-extract” demonstrated that three days post-treatment, “Nitric-extract” as such determined a significant reduction of rot severity over any other treatment in all fruits (oranges, lemons and apples). Additionally, “Nitric-extract” at 75% significantly reduced rot severity in lemon and apples over controls; “Nitric-extract” at 50% had a significant effect over controls only on apples; the concentration of 25% was as effective as the controls in all fruits. Five days post-treatment, “Nitric-extract” as such still maintained significant effects in reduction of rots only in lemons and apples; “Nitric-extract” at 75% demonstrated significant reduction of rot severity only in lemons; finally, “Nitric-extract” at 50% and at 25% were as effective as the controls in all tested fruits. Overall, the results showed an interesting performance of “Nitric-extract” in controlling postharvest mold caused by *Penicillium* spp., although the effective dose was much higher than that of traditional synthetic fungicides [7], and, as with other eco-friendly alternatives to synthetic fungicides [7,74], its use may not provide complete protection. A successful strategy for improving its efficacy or reducing fungicide residues from post-harvest fruit treatments could include the use of “Nitric-extract” in a mixture with conventional fungicides applied at a concentration lower than the standard dose, or by incorporating it in a fruit coating.

This study is part of a research program aimed at exploring the antifungal activity of extracts obtained from minimally processed shrimp wastes and their possible application in agriculture. The antifungal activity shown *in vitro* against a wide range of fungal and oomycete pathogens by the nitric extract appears promising and could be exploited in the context of new strategies for the management of plant diseases caused by these pathogens. *In vivo* preliminary results suggest a possible use of nitric extract for post-harvest treatments against citrus and apple molds caused by *Penicillium* species. To this aim, and to optimize the efficacy of treatments, next steps will be to define the methods and times of application. In this study, nitric extracts were applied to fruits 24 h after inoculation with the pathogen, indicating curative efficacy. However, an additional aspect that would merit further investigation is whether nitric extract, like other natural substances, is able to elicit plant defense mechanisms against infections by pathogens. In this case, the treatment of fruits with this extract might also have preventive efficacy against infections by molds. Regarding this, it cannot be ruled out that the other shrimp waste extracts, which, in preliminary *in vitro* tests did not show inhibitory activity on the mycelium growth, may also be effective *in vivo* acting as resistance elicitors. Last but not least, a prerequisite for the use of nitric extracts of shrimp waste to prevent post-harvest molds is to evaluate if the treatment leaves unpleasant odors on the fruits. A sensory analysis using an electron nose is planned to clarify this aspect. Although the effective dose of nitric shrimp waste extract is far higher than the label dose of synthetic fungicides used to control post-harvest fruit diseases, this extract, as a natural substance, could be an interesting alternative to traditional post-harvest chemical treatments, as it is more eco-friendly and far less toxic than synthetic fungicides.

## 4. Materials and Methods

### 4.1. Preparation of Shrimp Waste Substances

Around 5 kg of shrimp waste (cephalothorax, head and carapace) of the species *Parapenaeus longirostris*, common name deep-water rose shrimp, was collected in a local fish market in Catania (Italy), in February 2021. Shrimp waste was kept on ice until processed in the laboratory and firstly, washed with distilled water; then, dried in an oven at 30 °C for a week. The dried sample was powdered and homogenized. Then, 10 g of shrimp waste powder was packed in plastic food bags labelled “dry-powder” and stored at −20 °C until further use; 10 g of shrimp waste powder was processed to extraction (20 min long sonication by means of ArgoLab DU-100) with (i) 50 mL of ethyl acetate (EtOAc from Aldrich) or (ii) 50 mL of methanol (MetOH from Aldrich). Then, the supernatant was carefully collected and transferred into a clean beaker. The powder was then re-subjected to the described procedure for a total of three times. The 150 mL of supernatant were firstly filtrated and then evaporated under vacuum at the temperature of 40 °C until a crude extract was obtained. The crude extracts, representing the “EtOAc-extract” and “MetOH-extract”, were stored at −20 °C until further use. (iii) To obtain “Nitric-extract”, 20 mL of nitric acid (HNO3, 65% from Aldrich) was added to 5 g of shrimp waste powder; the mixture was then stirred for 1 h at 150 rpm and then the acid was neutralized by adding 80 mL of NaOH (0.10 g/mL). pH was verified to be around pH 5. To obtain the “water-extract”, 25 mL of water with 1% of acetic acid were added to 5 g of shrimp waste powder; the mixture was homogenized by vortexing and ultrasonication. The liquid extracts were then filtrated and stored at −20 °C until further use.

### 4.2. Analysis of Metabolites Present in Shrimp Waste Samples by HPLC-ESI-MS/TOF

The differential analysis of the metabolites contained in the four substances tested was carried out by HPLC-ESI-MS-TOF. Before the analysis, each sample was subjected to specific pretreatments. In particular, “EtOAc-extract” and “MetOH-extract” were dissolved in a methanol solution at 1% of acetic acid. Finally, “Water-extract” and “Nitric-extract” were mixed to acidified water. Each sample was finally filtered with 0.22 µm filter and then analyzed using an UPLC (1290 Infinity LC, Agilent Technologies, Santa Clara, CA, USA) coupled with a quadrupole time of flight mass spectrometer (Agilent 6546 LC/Q-TOF) operating in positive and negative ionization mode. Chromatographic separation was performed with an Agilent Zorbax RRHD SB-C18, 2.1 mm × 50 mm, 1.8 µm column. Mobile phase A was composed of Milli-Q water and acetonitrile was used for mobile phase B (both phases were acidified with 0.1% formic acid), with gradient elution, as follows: 0 min, 2% B; 22 min 95% B; 25 min, 5% B. The column was equilibrated for 3 min before every analysis. The flow rate was 0.4 mL/min, and 5 μL of sample was injected. Dual AJS ESI source conditions were as follows: gas temperature: 325 °C; gas flow: 10 L/min; nebulizer pressure: 40 psig; sheath gas temperature: 295 °C; sheath gas flow: 12 L/min; capillary voltage: 4000 V; nozzle voltage: 500 V; Fragmentor: 120 V; skimmer: 70 V; product ion scan range: 100–1500 Da; MS scan rate: 5 spectra/s; MS/MS scan rate: 3 spectra/s; maximum precursors per cycle: 2; and collision energy: 10, 20, 40 eV. The analysis of the metabolites was carried out in triplicate. Untargeted LC/Q-TOF based metabolomics approach was used to identify the metabolic profiling of shrimp waste extracts. Integration, data elaboration and identification of metabolites were managed using MassHunter Qualitative Analysis software B.08.00 and library PCDL Manager B.08.00.

### 4.3. Fungal and Oomycete Strains, Culture Conditions and Propagules Production

Fungal and oomycete strains were included in this study. Most of them had been previously characterized [7,8,55,79,80]. The complete list of strains tested in this study is as follows: four *Penicillium* spp. (*P. digitatum* P1PP0, *P. commune* CECT 20767, *P. expansum* CECT 2278 and *P. italicum* CECT 20909); three *Phytophthora* spp. (*Ph. nicotianae* strains T3-B-K1A and T2.C-M1A, *Ph. citrophthora* strain Ax1Ar); *Plenodomus tracheiphilus* strain Pt2; two *Alternaria* species (*A. alternata* strain 646, and *A. arborescens* strain 803); three *Colletotrichum* species (*C. acutatum* strains UW14, *C. karsti* strain CAM and *C. gloeosporioides* strain C2); two *Fusarium* species (*F. proliferatum* strain CBS 145950 and *F. sacchari* strain 145949). All strains were from the collection of the laboratory of Molecular Plant Pathology of the Di3A (University of Catania, Catania, Italy).

### 4.4. In Vitro Preliminary Screening for Selecting the Most Effective Extract

The antifungal activity of the “dry-powder”, “EtOAc-extract”, “MetOH-extract” and “Nitric-extract” were preliminarily checked in order to select, among them, the most promising one to be used in further tests.

For testing the effectiveness of the “dry-powder” in affecting mycelial growth, 16 g of shrimp waste powder were homogenized with 1 L of autoclaved PDA and poured in 90 mm Petri dishes. For each pathogen, a mycelial plug (diameter 3 mm) from a 7-day-old culture grown on PDA at 25 °C was transferred in the center of a “dry-powder”—amended PDA plate; control cultures of each pathogen, obtained by subcultures in “dry-powder”—non amended PDA plates, were included in the test. The plates were incubated at room temperature (20 ± 2 °C) for three days (for fungal pathogens) or for 15 days (for oomycete pathogens). At the end of the incubation period, no negative effects were observed in mycelial growth compared with controls for any of the pathogens. The “dry-powder” was not further tested.

The effect of “EtOAc-extract”, “MetOH-extract” and “Nitric-extract” on the mycelial growth of the pathogens was tested at different concentrations. To this purpose, “EtOAc-extract” and “MetOH-extract” were separately diluted in 1% dimethyl sulfoxide to obtain, for each substance, four solutions at the following concentrations 10, 25, 50 and 100 mg/mL; “Nitric-extract” was diluted in water to obtain the following concentrations: 25, 50, 75 and 100%.

“EtOAc-extract”, “MetOH-extract”, “Nitric-extract” and each fungal pathogen were tested separately in a 90 mm PDA plate as it follows: 500 µL of a suspension of conidia of the fungal pathogen (concentration 10^4^ conidia/mL) were homogeneously spread on the surface of a PDA plate; by using a cork borer, five wells (diameter 3 mm, each) were then realized on the PDA plate; then, 60 µL of each concentration of the substance were pipetted into the respective well; the plates were finally incubated at 25 °C for three days. For the oomycete pathogens (*Phytophthora* spp.), the influence of “EtOAc-extract”, “MetOH-extract” and “Nitric-extract” was tested separately as follows: for each *Phytophthora* strain, a mycelial plug (diameter 3 mm) from a 7-day-old culture grown on PDA at 25 °C was transferred in the center of a PDA plate and surrounded by 5 wells at a distance of 3 cm from the plug; then, 60 µL of each concentration of the substance tested were pipetted into the respective well. The plates were then incubated at 25 °C for 15 days.

In all the experiments, the possible mycelial growth inhibitory activity induced by each solvent used for the preparation of the respective extract was verified by *in vitro* tests performed as described above. For all pathogens and substances at each concentration, all the tests were performed in triplicate.

### 4.5. Minimum Inhibitory Concentration (MIC) and Minimum Fungicidal Concentration (MFC) of Nitric-Extract

The minimum inhibitory concentration (MIC) and the minimum fungicidal concentration (MFC) are dilution end points of a substance which completely inhibits the growth or kills the fungi tested; both are widely used in routine tests of substances with antimicrobial activity [9,81]. The minimum inhibitory concentration (MIC), defined as the lowest concentration of the test substance that inhibits visible growth, was determined with a microdilution method. For each pathogen, in a 2.0 mL tube, 400 µL of “Nitric-extract” at specific concentrations were added to 400 µL of sterile PDB and to 200 µL of spores suspension (concentration 10^4^ spores/mL) to obtain 10 serial dilutions (1 mL each) of the substance tested (final concentrations 0.5, 1.0, 1.5, 2.0, 2.5, 3.0, 3.5, 4.0, 4.5, 5.0%). Then, the tubes were incubated at 25 °C for 3 days.

After the incubation period, the MIC was the lowest concentration where no cloudiness was visible in the tubes, which means that no pathogen growth was observed. The determination of MFC was an additional step of the MIC test. The MFC is defined as the lowest concentration of a substance required to kill a fungal pathogen corresponding to no visible subculture growth on an unamended culture medium in environmental conditions favorable to the growth. In the present study, the evaluation of the MFC was carried out by transferring 10 μL from each of the wells where solution cloudiness was not observed into PDA medium. The inoculated plates were incubated at 25 °C for 3 days. The MFC for each pathogen was represented by the plated concentration that did not lead to any mycelial growth after the incubation period.

### 4.6. Evaluating the In Vivo Antifungal Activity of Nitric-Extract in Preventing Fruit Rots

The antifungal activity of the “Nitric-extract” was evaluated *in vivo* against infections caused by *P. digitatum* and *P. expansum* on citrus (oranges and lemons) and apple fruits, respectively.

#### 4.6.1. Nitric-Extract Dilutions

For the test, “Nitric-extract” was tested in all fruits (orange, lemon and apple) as such (ID02) or as three serial dilutions in sterilized distilled water (sdw) (concentrations; 75%—ID04; 50%—ID06; 25%—ID08). In addition, ID03, ID05, ID07 and ID09 were the respective controls.

In this experiment, four control groups were considered: (i) water (ID01); (ii) a solution of nitric acid (HNO3, 65%) and sodium hydroxide (NaOH, 0.1 g/mL) at the ratio 1:4—they are the solvent and base used for the preparation of “Nitric-extract”, respectively, which leads to a water solution of NaNO_3_ at the concentration 0.002 mol/mL (0.17 g/mL); (iii) NaNO_3_ 0.17 g/mL diluted in sdw at 75%—ID05; (iv) NaNO_3_ 0.17 g/mL diluted in sdw at 50%—ID07; NaNO_3_ 0.17 g/mL diluted in sdw at 25%—ID09 (Table 4).

#### 4.6.2. Fruits

All fruits used in this test came from organic crops. Citrus fruits were mature oranges (*Citrus* × *sinensis*) cv. Valencia and lemons (*Citrus* × *limon*) cv. Femminello Siracusano, while apples (*Malus domestica*) were of the cv. Braeburn. Before the tests, all fruits were preliminarily surface-disinfected by dipping in 1% NaClO (NaClO 0.5% for apples) for 2 min, rinsing under tap water and air-drying at room temperature (20 ± 2 °C).

#### 4.6.3. Fungal Pathogens and Inoculum Preparation

The strains used in the trial were *P. digitatum* strain P1PP0 and *P. expansum* strain CECT 2278. For each strain, the inoculum was represented by a conidial suspension at the concentration 10^6^ conidia/mL.

#### 4.6.4. Inoculation

Surface-disinfected fruits (oranges, lemons and apples) were wounded with a 2 mm-diameter plastic tip at four points along the equatorial surface; then, 10 µL of conidial suspension (*P. digitatum* strain P1PP0 for citrus and *P. expansum* strain CECT2278 for apples) was pipetted into each wound. Inoculated fruits were incubated in a plastic container at 20 °C and 80% RH (relative humidity) for 24 h. For all fruit (oranges, lemons and apples), the treatment with “Nitric-extract” as such or as a dilution was carried out as follows: after the incubation period, at each inoculation point, 20 µL of the substance was placed into the wound; overall, 3 fruits per treatment were used. An additional control group, represented by 3 fruits wounded as above, received 20 µL of sterile distilled water (sdw) per wound. The experiment was repeated another two times, with similar results. Analysis of variance did not reveal any differences among the experiments (F not significant); therefore, only the results of a single experiment are reported here.

#### 4.6.5. Evaluation of the Efficacy of the Nitric-Extract in Preventing Fruit Rot

The antifungal activity of “Nitric-extract”, as such, or as a dilution, was recorded at 3 and 5 days after inoculation and expressed as rot severity, rated according to empirical scales, from 1 to 5. This scale was different according to the fruit. For citrus fruits, the scale 25% was as follows: 1. absence of symptoms or signs of the pathogen; 2. slight presence of rot; 3. clear presence of rot and slight appearance of mycelium; 4. rot and clear presence of white mycelium; 5. clear presence of soft rot, white mycelium and sporulation. For apple fruits the scale was as follows: 1. absence of symptoms or signs of the pathogen; 2. slight presence of rot; 3. clear presence of rot and slight appearance of mycelium; 4. presence of rot, white mycelium and slight appearance of sporulation; 5. clear presence of soft rot, white mycelium and sporulation.

All data were subjected to one-way analysis of variance (ANOVA) using the R software (https://www.r-project.org/) (accessed on 9 November 2021). In order to normalize the distributions, data were transformed in square-root values, but untransformed values are reported in the respective graphs. Tukey’s HSD (honestly significant difference) post-hoc test was applied to evidence significant statistical differences (*p* ≤ 0.05).

## Figures and Tables

**Table 1:** Concentration of phenolic compounds detected in the tested substances (mean value ± standard deviation).

Phenolic Compounds (mg/kg)	Test Substances			
	Water-Extract	EtOAc-Extract	MetOH-Extract	Nitric-Extract
1-2-Dihydroxybenzene	Nd	0.48 ± 0.03	Nd	0.86 ± 0.02
3-(4-hydroxy-3-methoxyphenyl)	Nd	Nd	Nd	0.25 ± 0.03
Benzoic acid	2.48 ± 0.02	3.57 ± 0.04	3.08 ± 0.05	0.87 ± 0.02
Caffeic acid	0.16 ± 0.02	0.17 ± 0.03	0.58 ± 0.01	0.16 ± 0.04
Ellagic acid	Nd	Nd	Nd	0.20 ± 0.01
Gallic acid	Nd	Nd	Nd	Nd
Hydroxicinnamic acid	0.05 ± 0.03	0.42 ± 0.01	0.27 ± 0.02	Nd
P-Coumaric acid	0.27 ± 0.02	0.34 ± 0.02	0.83 ± 0.04	0.88 ± 0.01
Sinapic acid	0.10 ± 0.03	Nd	Nd	Nd
Syringic acid	0.16 ± 0.02	Nd	1.21 ± 0.03	0.22 ± 0.01
Vanillic acid	0.27 ± 0.01	Nd	Nd	0.56 ± 0.03
Vanillin	0.21 ± 0.02	0.37 ± 0.03	2.04 ± 0.03	0.36 ± 0.01

**Table 2:** Inhibitory effect of different concentrations (from 25 to 100%) of shrimp nitric-extract on the mycelium growth of 12 fungal and three oomycete plant pathogens, determined with the agar diffusion test by measuring the diameter of the inhibition halo around the wells. The incubation period was three days for fungi and 15 days for oomycetes.

	25% Nitric-Extract (Mean ± SD)	50% Nitric-Extract (Mean ± SD)	75% Nitric-Extract (Mean ± SD)	100% Nitric-Extract (Mean ± SD)
*Penicillium digitatum* P1PP0	14.00 ± 2.65 **c** ^1^; (*ab*) ^2^	20.00 ± 1.00 **b**; (*bc*)	25.00 ± 1.00 **a**; (*bcd*)	26.00 ± 1.00 **a**; (*de*)
*P. commune* CECT 20767	12.00 ± 1.73 **d**; (*bc*)	23.00 ± 0.00 **c**; (*ab*)	30.00 ± 1.00 **b**; (*bc*)	34.00 ± 1.73 **a**; (*b*)
*P. expansum* CECT 2278	0.00 ± 0.00 **d**; (*e*)	13.00 ± 1.73 **c**; (*ef*)	22.00 ± 0.00 **b**; (*cdef*)	27.00 ± 1.00 **a**; (*cde*)
*P. italicum* CECT 20909	0.00 ± 0.00 **d**; (*e*)	12.00 ± 1.73 **c**; (*f*)	20.00 ± 1.73 **b**; (*cdef*)	25.00 ± 0.00 **a**; (*def*)
*Colletotrichum acutatum* UW14	0.00 ± 0.00 **d**; (*e*)	15.00 ± 1.00 **c**; (*def*)	20.00 ± 0.00 **b**; (*cdef*)	22.00 ± 0.00 **a**; (*efg*)
*C. karsti* CAM	0.00 ± 0.00 **c**; (*e*)	11.00 ± 1.00 **b**; (*f*)	13.00 ± 1.00 **b**; (*f*)	19.00 ± 2.65 **a**; (*gh*)
*C. gloeosporioides* C2	12.00 ± 1.73 **c**; (*bc*)	15.00 ± 1.00 **c**; (*def*)	23.00 ± 1.73 **b**; (*cdef*)	32.00 ± 3.46 **a**; (*bc*)
*Fusarium proliferatum* CBS 145950	12.00 ± 1.00 **b**; (*bc*)	13.00 ± 2.65 **b**; (*ef*)	15.00 ± 1.00 **ab**; (*def*)	18.00 ± 1.73 **a**; (*gh*)
*F. sacchari* CBS 145949	15.00 ± 1.00 **c**; (*ab*)	17.00 ± 1.73 **c**; (*cde*)	21.00 ± 1.73 **b**; (*cdef*)	27.00 ± 0.00 **a**; (*cde*)
*Alternaria arborescens* 803	0.00 ± 0.00 **c**; (*e*)	12.00 ± 1.73 **b**; (*f*)	18.00 ± 1.73 **a**; (*def*)	20.00 ± 1.73 **a**; (*fgh*)
*A. alternata* 646	14.00 ± 1.00 **c**; (*ab*)	19.00 ± 1.00 **b**; (*bcd*)	24.00 ± 1.00 **a**; (*bcde*)	25.00 ± 1.73 **a**; (*def*)
*Plenodomus tracheiphilus* Pt2	10.00 ± 2.00 **d**; (*c*)	15.00 ± 1.73 **c**; (*def*)	34.00 ± 1.00 **b**; (*a*)	43.00 ± 1.00 **a**; (*a*)
*Ph. nicotianae* T2.C-M1A	16.00 ± 1.73 **d**; (*a*)	26.00 ± 1.00 **c**; (*a*)	34.00 ± 0.00 **b**; (*ab*)	30.00 ± 2.65 **a**; *(bcd*)
*Ph. nicotianae* T3-B-K1A	4.00 ± 1.73 **d**; (*d*)	12.00 ± 2.00 **c**; (*f*)	16.00 ± 1.00 **b**; (*def*)	20.00 ± 1.73 **a**; (*fgh*)
*Ph. citrophthora* Ax1Ar	0.00 ± 0.00 **c**; (*e*)	12.00 ± 0.00 **b**; (*f*)	14.00 ± 1.73 **ab**; (*ef*)	16.00 ± 1.00 **a**; (*h*)

^1^ In a horizontal direction, for each pathogen, values with different bold letters are statistically different according to Tukey’s honestly significant difference (HSD) test (*p* ≤ 0.05). ^2^ In the vertical direction, for the concentrations 25% Nitric-extract, 50% Nitric-extract, 75% Nitric-extract, 100% Nitric-extract, values with different letters (in *italic* and within brackets) are statistically different according to Tukey’s honestly significant difference (HSD) test (*p* ≤ 0.05).

**Table 3:** Minimum inhibitory concentration (MIC) and minimum fungicidal concentration (MFC) determined by the nitric-extract.

	Nitric-Extract (%)	
Pathogen (Species, Strains)	MIC	MFC
*Penicillium digitatum* P1PP0	3.0	3.0
*P. commune* CECT 20767	2.5	3.0
*P. expansum* CECT 2278	3.5	3.5
*P. italicum* CECT 20909	3.0	3.0
*Colletotrichum acutatum* UW14	2.5	2.5
*C. karsti* CAM	3.0	3.0
*C. gloeosporioides* C2	2.0	2.0
*Fusarium proliferatum* CBS 145950	3.0	3.0
*F. sacchari* CBS 145949	3.5	3.5
*Alternaria arborescens* 803	2.5	2.5
*A. alternata* 646	2.5	3.0
*Plenodomus tracheiphilus* Pt2	2.0	2.0
*Phytophthora nicotianae* T2.C-M1A	2.5	2.5
*P. nicotianae* T3-B-K1A	2.0	2.5
*P. citrophthora* Ax1Ar	2.5	3.0

**Table 4:** List of treatments of the *in vivo* tests with shrimp powder extract (Nitric-extract). Control IDs were obtained by adding sterile distilled water (sdw) to NaNO_3_ (0.17 g/mL).

ID of Treatment	Tested Substance
ID01	WATER
ID02	100% Nitric-extract (NaNO_3_ 0.17 g/mL)
ID03	NaNO_3_ 0.17 g/mL
ID04	75% Nitric-extract
ID05	NaNO_3_ 0.17 g/mL diluted in sdw at 75%
ID06	50% Nitric-extract diluted in sdw
ID07	NaNO_3_ 0.17 g/mL diluted in sdw at 50%
ID08	25% Nitric-extract
ID09	NaNO_3_ 0.17 g/mL diluted in sdw at 25%

## Data Availability

Raw data can be shared upon reasonable request.
